# Supplementary material for: Attachment style and parental bonding: Relationships with fibromyalgia and alexithymia
Source: PLoS One. 2020 Apr 14;15(4):e0231674. doi: 10.1371/journal.pone.0231674 (PMC7156042; doi:10.1371/journal.pone.0231674)
Supplement: Attachment — Submitted filename: Response to Reviewers.docx [file pone.0231674.s002.docx]

February 4, 2020

Dear Juan V. Luciano,

please find below a point by point response to all your comments. Thank you for the opportunity to clarify the issues that you raised.

1. *Please provide additional information about the participant recruitment method and the demographic details of your participants. Please add in your methods section such details as a) the recruitment date range (month and year), b) a statement as to whether your sample can be considered representative of a larger population, c) a description of how participants were recruited, and d) descriptions of where participants were recruited and where the research took place.*

**Following your indications, we added the required information. Specifically, we added the recruitment date range, the recruitment details for both FM patients and HC. Our FM patients were recruited consecutively, so we added the following sentence in the text: “Patients were recruited consecutively; therefore, the resulting sample is more likely to represent the target population than one resulting from simple convenience sampling.”**

*2. Internal editors noticed that the present submission is closely related to previously published works, including:*

- *Di Tella MD, Enrici I, Castelli L, Colonna F, Fusaro E, Ghiggia A, Romeo A, Tesio V, Adenzato M. Alexithymia, not fibromyalgia, predicts the attribution of pain to anger-related facial expressions. J Affect Disord. 2018; 227: 272-279.*
- *Di Tella M, Tesio V, Ghiggia A, Romeo A, Colonna F, Fusaro E, Geminiani GC, Bruzzone M, Torta R, Castelli L. Coping strategies and perceived social support in fibromyalgia syndrome: Relationship with alexithymia. Scand J Psychol. 2018; 59(2): 167-176.*
- *Ghiggia A, Romeo A, Tesio V, Tella MD, Colonna F, Geminiani GC, Fusaro E, Castelli L. Alexithymia and depression in patients with fibromyalgia: When the whole is greater than the sum of its parts. Psychiatry Res. 2017 Sep;255:195-197.*
- *Di Tella M, Ghiggia A, Tesio V, Romeo A, Colonna F, Fusaro E, Torta R, Castelli L. Pain experience in Fibromyalgia Syndrome: The role of alexithymia and psychological distress. J Affect Disord. 2017 Jan 15;208:87-93.*
- *Tesio V, Di Tella M, Ghiggia A, Romeo A, Colonna F, Fusaro E, Geminiani GC, Castelli L. Alexithymia and Depression Affect Quality of Life in Patients With Chronic Pain: A Study on 205 Patients With Fibromyalgia. Front Psychol. 2018; 9: 442.*

*Please ensure that the related works have been adequately discussed, by commenting both in your cover letter and in the manuscript on how this work constitutes a distinct contribution.*

*Please also ensure that any overlap in the dataset and analyses with previous works has not compromised the robustness of the statistical analysis (e.g., by removing required adjustments for multiple hypothesis testing). For further information on our submission guidelines on related manuscripts, please see* [*http://journals.plos.org/plosone/s/submission-guidelines#loc-related-manuscripts*](http://journals.plos.org/plosone/s/submission-guidelines#loc-related-manuscripts)*.*

**Following your remarks, we discussed the specificity of this paper with respect to our previous studies on FM patients both in the manuscript (discussion section) and in the opening lines of the cover letter.**

**Furthermore, we specified that the current dataset has not been used for other studies (we added the following sentence in the text: “No previous study has been published yet, using the present dataset”). We carried out different studies investigating the psychological components of FM patients, but for each study we enrolled different patients in different periods of time.**

Sincerely,

Annunziata Romeo, Ph.D.

Department of Psychology, University of Turin

Via Verdi, 10, 10124, Turin, Italy

Email address: [annunziata.romeo@unito.it](mailto:annunziata.romeo@unito.it)
